# The impact of ketamine and thiopental anesthesia on ultraweak photon emission and oxidative-nitrosative stress in rat brains

**DOI:** 10.3389/fnsys.2025.1502589

**Published:** 2025-03-21

**Authors:** Mahdi Khorsand Ghaffari, Niloofar Sefati, Tahereh Esmaeilpour, Vahid Salari, Daniel Oblak, Christoph Simon

**Affiliations:** ^1^Department of Physiology, School of Medicine, Shiraz University of Medical Sciences, Shiraz, Iran; ^2^Department of Anatomy, School of Medicine, Arak University of Medical Sciences, Arak, Iran; ^3^Department of Anatomical Sciences, School of Medicine, Shiraz University of Medical Sciences, Shiraz, Iran; ^4^Department of Physics and Astronomy, University of Calgary, Calgary, AB, Canada; ^5^Institute for Quantum Science and Technology, University of Calgary, Calgary, AB, Canada; ^6^Hotchkiss Brain Institute, University of Calgary, Calgary, AB, Canada

**Keywords:** anesthesia, ketamine, thiopental, brain isolation, ultraweak photon emission (UPE)

## Abstract

Anesthetics such as ketamine and thiopental, commonly used for inducing unconsciousness, have distinct effects on neuronal activity, metabolism, and cardiovascular and respiratory systems. Ketamine increases heart rate and blood pressure while preserving respiratory function, whereas thiopental decreases both and can cause respiratory depression. This study investigates the impact of ketamine (100 mg/kg) and thiopental (45 mg/kg) on ultraweak photon emission (UPE), oxidative-nitrosative stress, and antioxidant capacity in isolated rat brains. To our knowledge, no previous study has investigated and compared UPE in the presence and absence of anesthesia. Here, we compare the effects of ketamine and thiopental anesthetics with each other and with a non-anesthetized control group. Ketamine increased UPE, lipid peroxidation, and antioxidant enzyme activity while reducing thiol levels. Conversely, thiopental decreased UPE, oxidative markers, and antioxidant enzyme activity, while increasing thiol levels. UPE was negatively correlated with thiol levels and positively correlated with oxidative stress markers. These findings suggest that the contrasting effects of ketamine and thiopental on UPE are linked to their differing impacts on brain oxidative stress and antioxidant capacity. This research suggests a potential method to monitor brain oxidative stress via UPE during anesthesia, and opens up new ways for understanding and managing anesthetic effects.

## Graphical abstract

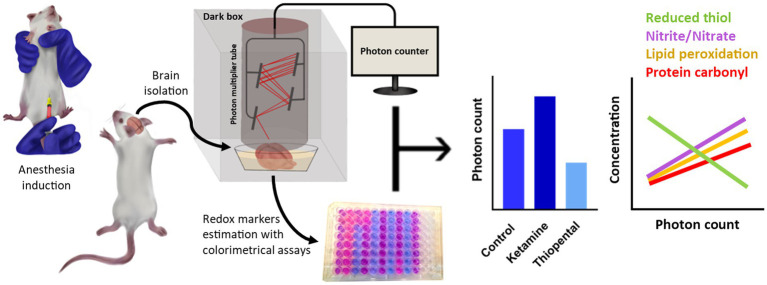

## 1 Introduction

Anesthetics induce regulated unconsciousness associated with extensive brain network connectivity perturbations. They are used for analgesia, sedation, and hypnosis during surgery and in the intensive care unit. The mechanism of anesthesia is still not fully understood. This may be due to the ilusive nature of consciousness (Hemmings et al., 2019). Several neuroscientific theories of consciousness have been proposed and are currently the subject of heated debate. Although these ideas may seem different at first glance, they are all fundamentally rooted in various aspects of interconnectedness. Their shared belief is that the richness of neuronal interactions in corticothalamic systems is crucial for achieving consciousness. Specifically, when interacting neurons reach a critical level of complexity, a conscious experience is formed (Storm et al., 2024).

Large-scale neural oscillations are intricately linked to essential cognitive functions, including perception, attention, decision-making, memory, and consciousness (Plankar et al., 2013). In a study, it has been shown that different spectral light stimulations at one end of the spinal sensory or motor nerve roots significantly increased the biophotonic activity at the other end (Sun et al., 2010). Thus, local anesthetic or metabolic inhibitors may inhibit these effects. Ultraweak photon emission (UPE) is closely linked to reactive oxygen species (ROS), with intensity variations reflecting various physiological and pathological states, such as stress (thermal, chemical, mechanical), mitochondrial function, cell cycle, etc., and UPE intensity correlates with neural activity, oxidative reactions, EEG activity, cerebral blood flow, energy metabolism, and glutamate release (Esmaeilpour et al., 2020; Isojima et al., 1995; Kobayashi et al., 1999). Different anesthetics produce diverse EEG signatures linked to their molecular targets and affected neural circuits. For example, Thiopental dose-dependently decreases EEG frequency until the EEG becomes isoelectric, while ketamine increases high-frequency gamma waves (MacIver et al., 1996; Arena et al., 2022). High-frequency oscillations are linked to neuronal firing and cortical activation, while low-frequency oscillations occur during the resting state (Poulet and Crochet, 2019). Therefore, anesthetics and their effect on UPE might be somehow correlated with brain function, EEG, and cognitive behaviors (Salari et al., 2022; Salari et al., 2016).

Neural communication depends on the release of neurotransmitters. This intricate process involves the transportation of molecules to presynaptic active zones by motor proteins, regulation of ion channel function, mobilization of synaptic vesicles, and recycling of neurotransmitters. Each of these steps requires energy in the form of ATP, and general anesthetics can impact these essential processes (Bademosi et al., 2018; Baumgart et al., 2015; Bensel et al., 2017). The predominant energy expenditure in the brain involves maintaining ion gradients across cellular membranes, which is largely fuelled by the oxidative metabolism of glucose. The energy demands of glutamatergic neurons account for 80–90% of cortical glucose consumption (Magistretti et al., 1999). Astrocytes primarily remove released glutamate from the synaptic cleft. A study has shown a close coupling between cerebral glucose metabolism and the cycling of glutamate between neurons and astrocytes (Sibson et al., 1998).

Anesthesia induction with ketamine and thiopental has different effects on glutamatergic transmission. Ketamine inhibits NMDA receptors of cortical interneurons more efficiently than those on pyramidal neurons. This inhibition leads to reduced interneuron activity and subsequently decreases the release of GABA on pyramidal neurons. The reduction in GABA release ultimately results in increased glutamate release and heightened excitation of pyramidal neurons. Additionally, ketamine may interrupt the steady inhibition of glutamate release by inhibiting presynaptic NMDA receptors (Moghaddam et al., 1997; McMillan and Muthukumaraswamy, 2020). Thiopental is a GABA_A_ receptor agonist which depresses cerebral activity by prolonging the opening of the Cl-channel and hyperpolarization induction. Moreover, it has been shown that thiopental sodium inhibits glutamate release from cerebrocortical slices (Ito et al., 1996; Miao et al., 1998). These aforementioned mechanisms explain why ketamine heightens cerebral glucose metabolism while thiopental decreases it (Långsjö et al., 2004; Sokoloff et al., 1977).

UPE has been detected during the cellular metabolism of all living systems. UPE arises from the electronic relaxation of excited species formed during reactions of reactive oxygen species (ROS) and reactive nitrogen species (RNS) and their derivatives (Pospíšil et al., 2014). The interaction of biomolecules (lipids, proteins, nucleic acids) with these reactive species creates high-energy intermediate products that eventually lead to the formation of singlet or triplet excited state carbonyl compounds and singlet ground state oxygen. When these species undergo electronic transitions, photons are emitted at short and long wavelength regions of the spectrum, respectively (Pospíšil et al., 2019).

ROS and RNS primarily originate from the respiratory chain of mitochondria, NADPH oxidases, nitric oxide synthases, xanthine oxidase, cytochrome P450 oxidases, lipoxygenases, and cyclooxygenases. Respiration generates oxygen-centered free radicals, which are molecules with unpaired electrons. The reduction of molecular oxygen to water involves the addition of four electrons in several steps. Molecular oxygen (O_2_) is first reduced to form the superoxide anion radical (O_2_^−^), which then reacts with its conjugate acid to produce hydrogen peroxide (H_2_O_2_). Hydrogen peroxide is a two-electron reduction product of O_2_ and serves as both a byproduct and a source of free radical reactions. It can also react with iron or other transition metals to produce the hydroxyl radical (OH), a powerful oxidant. Additionally, Superoxide can react with nitric oxide (NO) to form the peroxynitrite anion (OONO^−^). When protonated to peroxynitrous acid (OONOH), it decomposes to yield the hydroxyl radical (Dröge, 2002). Superoxide dismutase (SOD) facilitates the conversion of O_2_^−^ into H_2_O_2_. The catalase enzyme (CAT) subsequently breaks down H_2_O_2_ into H_2_O and O_2_, or it can be transformed by thiol peroxidases, such as glutathione peroxidase, which promote the reduction of H_2_O_2_ and/or organic hydroperoxides into water and the corresponding alcohols. Unlike SOD and CAT, peroxidases utilize a thiol-based reaction mechanism to neutralize hydroperoxides. Thiol reduced state must be replenished by reductases, such as glutathione reductase, with the consumption of NADPH (Flohé et al., 2011).

Thiol is an organosulfur compound in the form R−SH, where R represents an alkyl or other organic substituent, and −SH is a functional sulfhydryl group. Cysteine amino acid represents this characteristic. Organisms create millimolar concentrations of cysteine-containing thiols that serve as a cofactor for thiol-dependent enzymes. Moreover, they form covalent linkages with protein thiols to protect against overoxidation and reduce existing oxidative modifications in proteins. The tripeptide *γ*-glutamyl cysteinyl glycine, known as glutathione (GSH), appears to maintain cellular redox homeostasis in most eukaryotes (Ulrich and Jakob, 2019). When ROS are effectively scavenged by GSH, their oxidative effects on biomolecules are prevented. However, biomolecules are oxidized if ROS formation exceeds the antioxidant defence system’s capacity. Studies have shown that the addition of exogenous GSH, SOD, and CAT suppressed ultra-weak photon emission (Rastogi and Pospíšil, 2011; Kakinuma et al., 1979).

Ketamine and thiopental anesthetics have the opposite effect on brain metabolism when assessed using neuroimaging techniques. However, these methods may be affected by respiratory and cardiovascular interference (Murphy et al., 2013). Studies have shown that ketamine and thiopental have opposite respiratory and cardiovascular effects (Slupe and Kirsch, 2018). Isolated brain involves submerging the brain in oxygenated artificial cerebrospinal fluid. This approach maintains brain integrity without perfusing the vascular system. The viability of the rat brain using this method was confirmed by the successful recording of extracellular field potentials 1 day later (Von Bohlen and Halbach, 1999). Moreover, the EEG activity of the isolated brain was recorded and could last for up to 30 min (Gottschalk et al., 2019).

To our knowledge, no previous study has investigated and compared UPE in the presence and absence of anesthesia. Given that UPE is closely linked to oxidative metabolism and considering that anesthesia can alter brain metabolism and activity, we examine the isolated brain’s UPE under anesthesia with ketamine and thiopental. We also evaluate the relationship between changes in UPE and the brain’s oxidative-nitrosative state and antioxidant capacity.

## 2 Materials and methods

### 2.1 Drugs and reagents

Ketamine (Vetased^R^, Pasteur, Romania), Thiopental, Ethylenediaminetetraacetic acid (EDTA), thiobarbituric acid (TBA), 1,1,3,3-tetraethoxypropane (TEP), vanadium (III) chloride (VCL3), sulfanilamide, N-(1-Naphthyl) ethylenediamine dihydrochloride (NEDD), sodium nitrite (NaNO2), 2,4-dinitrophenylhydrazine (DNPH), trichloroacetic acid (TCA), guanidine hydrochloride, 5,5-Dithiobis-2-nitrobenzoic acid (DNTB). Except for ketamine, all other chemicals were purchased from Sigma-Aldrich.

### 2.2 Artificial cerebrospinal fluid (aCSF) composition

The aCSF contains 124 mM NaCl, 3 mM KCl, 26 mM NaHCO_3_, 1.25 mM Na_2_HPO_4_, 1.8 mM MgSO_4_, 1.6 mM CaCl_2_, 10 mM glucose.

### 2.3 Animals

Eighteen male Sprague–Dawley (SD) rats weighing between 180 and 190 g were randomly assigned to three experimental groups: Control, Ketamine, and Thiopental (*n* = 6). Rats were purchased from the Comparative and Experimental Medical Center of the Shiraz University of Medical Sciences (SUMS). All rats were kept in standard conditions, including a temperature of 22 ± 2°C, relative humidity of 50%, and a 12-h light/dark cycle, with free access to laboratory food and water. Animal procedures complied with the National Institutes of Health’s Guide for the Care and Use of Laboratory Animals and the ARRIVE Guidelines. The university’s Ethics Committee approved the procedures (approval number: IR.SUMS.REC.1400.191).

### 2.4 Study design

The control group received an intraperitoneal (IP) injection of 5 mL/kg of saline. After 3 min, the head was dislocated, and the brain was quickly removed to minimize any oxidative and anesthetic interventions. Other rats were given IP ketamine or thiopental. Following injection, anesthesia induction was checked with the loss of three successive righting reflexes. Then, their heads were dislocated, and the brain was rapidly removed. A study found that Sprague–Dawley rats had similar anesthesia induction and maintenance times with IP injection of 100 mg/kg ketamine or 45 mg/kg thiopental (Kushikata et al., 2011). Therefore, these doses were selected for comparison in the present study. After the brain was removed, it was flooded into a chamber containing fresh oxygenated aCSF (95% O_2_, 5% CO_2_). To prevent any possible delayed luminescence, the chamber was left in a dark room for 10 min while being continuously oxygenated with carbogen at room temperature (Sefati et al., 2024). After that, the brain was transferred to a chamber filled with fresh oxygenated aCSF at room temperature (~30°C) and positioned under a photomultiplier tube (PMT) to detect UPE for 300 s. Next, the brain was fast-frozen with liquid nitrogen and stored at −80 for further assessment of reduced thiol, lipid peroxidation, nitrite/nitrate levels, protein carbonyl, and antioxidant enzyme activity (SOD, CAT).

### 2.5 Brain extraction procedure

In this study, a recently published method was used for the rapid and intact extraction of rat brains (Aboghazleh et al., 2024). After cervical dislocation, the cervical spine was severed from the cranium. The scalps of the severed heads were incised mid-sagittally and retracted on both sides to reveal the skull. The temporalis muscles were removed on both sides. An incision was made through the pharyngeal muscles and those near the skull base; the mandible’s ramus was dislocated and taken out. The skull’s tympanic bullae were removed. After each step, the skull was rinsed in ice-cold PBS to cool and remove debris. For brain exposure first, the occipital bones around the foramen magnum and the cerebellum were dissected. Subsequently, the temporal bones of each side were taken out. After that, the parietal and frontal bones covering the cortex were removed and the olfactory bulbs were dissected. The trigeminal and optic nerves were cut at the inferior surface of the brain, and finally, the brain was extracted. The procedure lasted under 15 min from the anesthesia induction to the start of UPE monitoring.

### 2.6 UPE detection

In this study, a PMT (R6095 Hamamatsu Photonics K.K., Japan) was used to detect UPE in a dark box. The PMT has a quantum efficiency (QE) that varies between 20 and 30% in the range 300–700 nm, with the maximum QE at 420 nm. PMT amplifies entrance photons to electrical signals in a field of view. The analog signals were converted to digital using an RS485 to RS232 converter and then connected to a laptop for recording. The collecting gate time from the PMT was set to 1 s. Noise is reduced by modifying the upper and lower thresholds via PMT software. Dark noise was detected in an empty box for 5 min (~1,100 counts per 5 min) and subtracted from the results. The distance between the sample and the PMT sensor was adjusted to 0.5 cm. Before each trial, the aCSF emissions were recorded for 5 min and subtracted from sample emissions.

### 2.7 Redox markers assessment

#### 2.7.1 Brain homogenate preparation

The frozen brain was weighed and then homogenized using a Homogenizer (T 10 basic ULTRA-TURRAX, IKA, Germany) in ice-cold EDTA-potassium phosphate buffer for around 3 min. After homogenization, the mixture was centrifuged at 12,000 rpm at 4°C for 5 min. The resulting supernatant was then separated and stored at −80°C for further colorimetric measurements with a microplate reader (BioTek Synergy H1, Agilent, USA).

#### 2.7.2 Estimation of lipid peroxidation level

The level of lipid peroxidation in animal tissues was measured using the TBA reaction method. In this method, the pink color produced during the reaction of TBA with peroxidized lipids was measured at 532 nm for the estimation of lipid peroxidation, and the TEP was used as a standard (Ohkawa et al., 1979). TBA stands for “thiobarbituric acid,” a chemical used in the “thiobarbituric acid reactive substances (TBARS)” assay to measure lipid peroxidation. The TBA reacts with malondialdehyde (MDA), a byproduct of lipid peroxidation, forming a pink chromogen that can be quantified by measuring absorbance at 532 nm. TEP stands for “1,1,3,3-Tetraethoxypropane,” which is commonly used as a standard in lipid peroxidation assays like the TBARS assay. It is used because TEP can generate MDA when hydrolyzed, which then reacts with TBA to form the pink chromogen measured at 532 nm. By comparing the absorbance of sample to that of known TEP standards, one can quantify the amount of lipid peroxidation in your samples.

#### 2.7.3 Estimation of nitrite/nitrate level

The nitrite/nitrate level is estimated based on the previously described colorimetric method (Miranda et al., 2001). First, nitrate is converted to nitrite with a reaction to VCL3. Subsequently, the nitrite reacts with sulfanilamide at low pH to produce a diazonium salt. This salt is then reacted with NEDD to form a stable compound. The nitrite/nitrate level of the sample is estimated by comparing the absorbance of this compound with a standard curve of NaNO2 at 540 nm.

#### 2.7.4 Estimation of protein carbonyl

The protein’s oxidative damage was assessed by measuring the presence of carbonyl groups, which could be estimated by reacting them with DNPH. As described by Levine et al. (1990), DNPH in hydrochloride was added to the homogenate supernatant and incubated for 1 h. Subsequently, proteins were precipitated by adding TCA. After centrifugation, the supernatant was removed, and the pellets were washed with ethanol and ethyl acetate to eliminate excess DNPH. They were then dissolved in a solution of guanidine hydrochloride, and the absorbance was measured at 375 nm. Protein carbonyl level was determined using a molar extinction coefficient of 22,000 M−1 cm^−1^.

#### 2.7.5 Estimation of reduced thiol concentration

The concentration of reduced thiol was determined by a colorimetric method. In this method, Ellman’s reagent, also known as DNTB, reacts with reduced sulfhydryl groups. The complex formed is called the 5-thionitrobenzoic acid chromophore. This complex produces a yellow color, and the absorbance at 405 nm is used to estimate the concentration of reduced thiol in the sample (Rahman et al., 2006).

#### 2.7.6 Superoxide dismutase and catalase activity estimation

SOD and CAT activity were measured using the colorimetric method, according to the manufacturer’s instructions of the Assay Kit (ZellBio GmbH, Germany).

#### 2.7.7 Supernatant protein concentration

The Bradford method was used to assess the total protein content in the tissue supernatant (Kruger, 2009). Molecular data were expressed as x/mg protein of the supernatant to normalize the concentration.

### 2.8 Statistical analysis

Data were expressed as the means ± standard error of the mean (SEM). Statistical significance was set at *p* < 0.05. A one-way ANOVA followed by Tukey’s *post-hoc* test was used to compare the results among different groups. The relationship between UPE and oxidative-nitrosative stress was evaluated by Pearson correlation analysis. All statistical analyses were conducted using GraphPad version 8 (Prism Software Inc., San Diego, CA, USA).

## 3 Results

### 3.1 Effects of anesthesia induction on brain UPE

**Figure 1:**
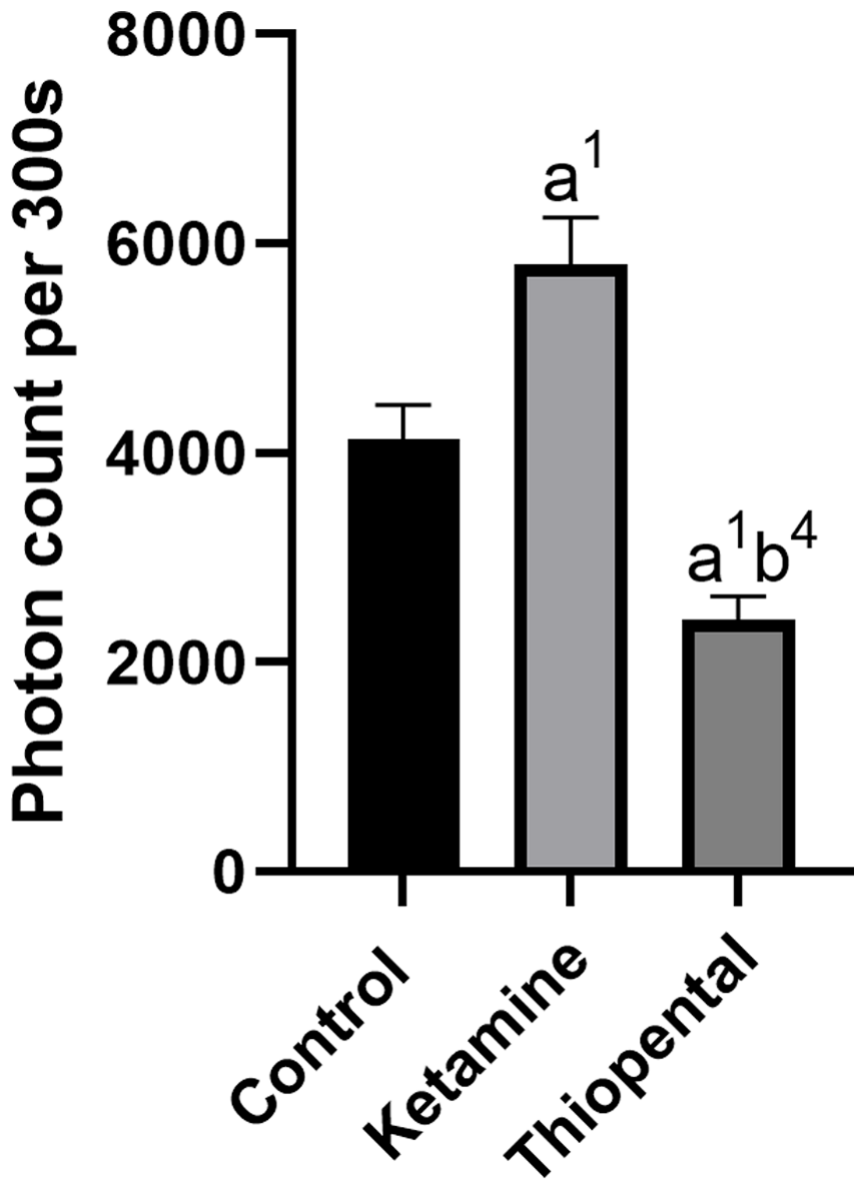
Number of brain’s UPE counted in the 300 s. Data were presented as mean ± SEM (*n* = 6) and analyzed by one-way ANOVA followed by Tukey’s multiple comparison test. (a) Compared to the control group, and (b) compared to the ketamine group. ^1^*p* < 0.05, ^4^*p* < 0.0001.

In this study, we found that anesthesia induction significantly altered the UPE of the isolated brain. Specifically, ketamine increased UPE while thiopental decreased it when compared to the control group (*p* < 0.05; Figure 1). In comparison between anesthesia groups, the thiopental group had lower brain UPE than the Ketamine group (*p* < 0.0001).

### 3.2 Effects of anesthesia induction on brain oxidative and nitrosative state

**Figure 2:**
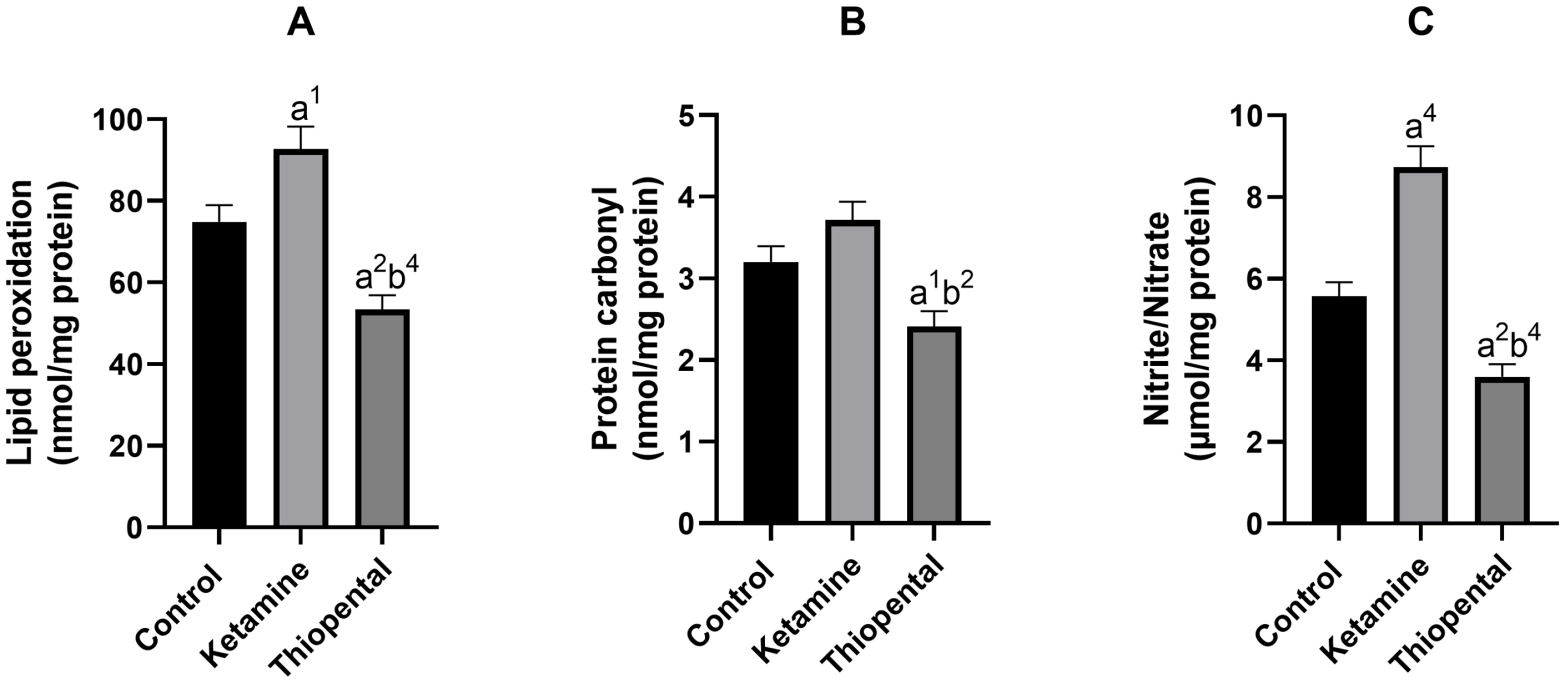
Brain oxidative and nitrosative state. **(A)** Lipid peroxidation level of the brain, **(B)** protein carbonyl content of the brain, **(C)** nitrite/nitrate level of the brain. Data were presented as mean ± SEM (*n* = 6) and analyzed by one-way ANOVA followed by Tukey’s multiple comparison test. (a) Compared to the control group, and (b) compared to the ketamine group. ^1^*p* < 0.05, ^2^*p* < 0.01, ^4^*p* < 0.0001.

Results from the oxidative and nitrosative state of the isolated brain showed that ketamine significantly increased lipid peroxidation (Figure 2A) and nitrite/nitrate level (Figure 2C) as compared to the control group (*p* < 0.05, *p* < 0.0001, respectively). In contrast, thiopental decreased the lipid (*p* < 0.01; Figure 2A), protein (*p* < 0.05; Figure 2B) and nitrogen (*p* < 0.01; Figure 2C) oxidation of the brain as compared to the control. In comparison between anesthesia groups, the thiopental group had a significantly lower brain oxidation state than the ketamine group (*p* < 0.01; Figure 2).

### 3.3 Changes in the brain’s antioxidant capacity under anesthesia

**Figure 3:**
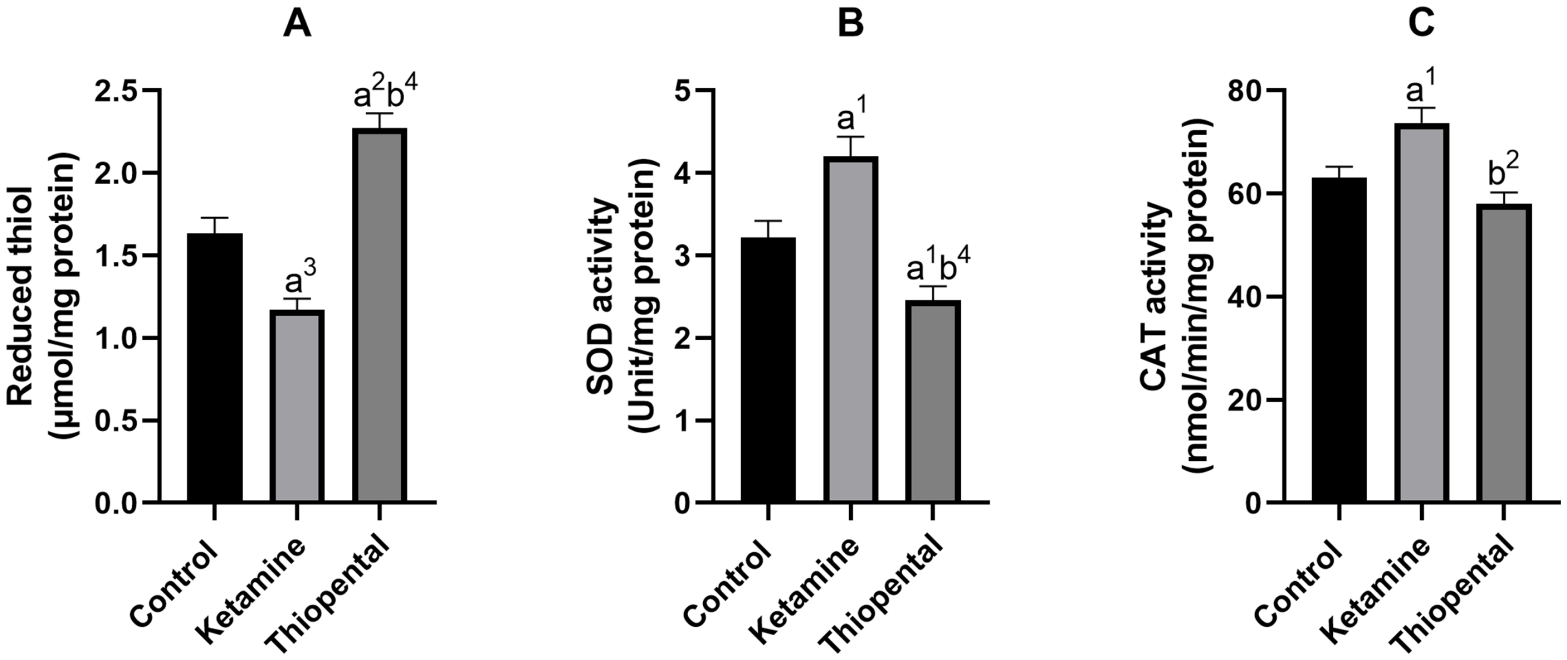
Antioxidant capacity of the brain. **(A)** Reduced thiol level of the brain, **(B)** SOD activity of the brain, **(C)** CAT activity of the brain. Data were presented as mean ± SEM (*n* = 6) and analyzed by one-way ANOVA followed by Tukey’s multiple comparison test. (a) Compared to the control group, and (b) compared to the ketamine group. ^1^*p* < 0.05, ^2^*p* < 0.01, ^3^*p* < 0.001, ^4^*p* < 0.0001.

As shown in Figure 3A, the brain reduced thiol level of the ketamine group was significantly lower than the control (*p* < 0.001). Brain antioxidant enzyme activity assessment indicated that anesthesia induction with ketamine increased brain SOD (Figure 3B) and CAT (Figure 3C) activity as compared to the control (*p* < 0.05). In contrast, anesthesia induction with thiopental increased reduced thiol level and decreased SOD activity compared to the control (*p* < 0.01, *p* < 0.05, respectively). In comparison between anesthesia groups, the thiopental group had higher reduced thiol (*p* < 0.0001; Figure 3A) and lower SOD (*p* < 0.0001; Figure 3B) and CAT activity (*p* < 0.01; Figure 3C) than the ketamine group.

## 4 Discussion

In this study, we try to replicate the method of previous studies which isolated the rodent brain and validated their viability. The brain viability could be validated by field potential, EEG, neuro tracers, and calcium imaging. We were unable to assess viability using these techniques because they required fluorescent illumination that interfered with UPE detection. Additionally, the PMT was positioned close to the brain, as it operates at high voltage and has a powerful cooling system. As a result, the electrical signals became noisy and undetectable. Electrical stimulation of isolated and perfused guinea pig brains (via the cortical vasculature) produced field potentials like those seen *in vivo* for up to 8 h. In this method, brains dissected within 8–15 min showed good viability (Mühlethaler et al., 1993). Extracellular field potentials and transport of neurotracer were successfully recorded in isolated mouse and rat brains for up to 24 h, without perfusion. In this method, a few necrotic cells were seen in rat brains which it overcame by dividing the brain into two hemispheres 30 min after extraction (Von Bohlen and Halbach, 1999). Another study validated neural viability and functionality of isolated mouse brains by calcium imaging and EEG monitoring at room temperature for up to 30 min, without perfusion (Gottschalk et al., 2019). In the studies mentioned above, as in the present study efforts were made to extend viability by reducing brain metabolic demand through cooling during brain extraction. So there is strong evidence suggesting that the brain can remain functional and viable for at least 30 min after being extracted. Since the total duration from the extraction to the UPE monitoring did not exceed this timeframe, we can assume that the data collected during our experiment reflects neuronal functionality similar to that observed in a living brain.

**Figure 4:**
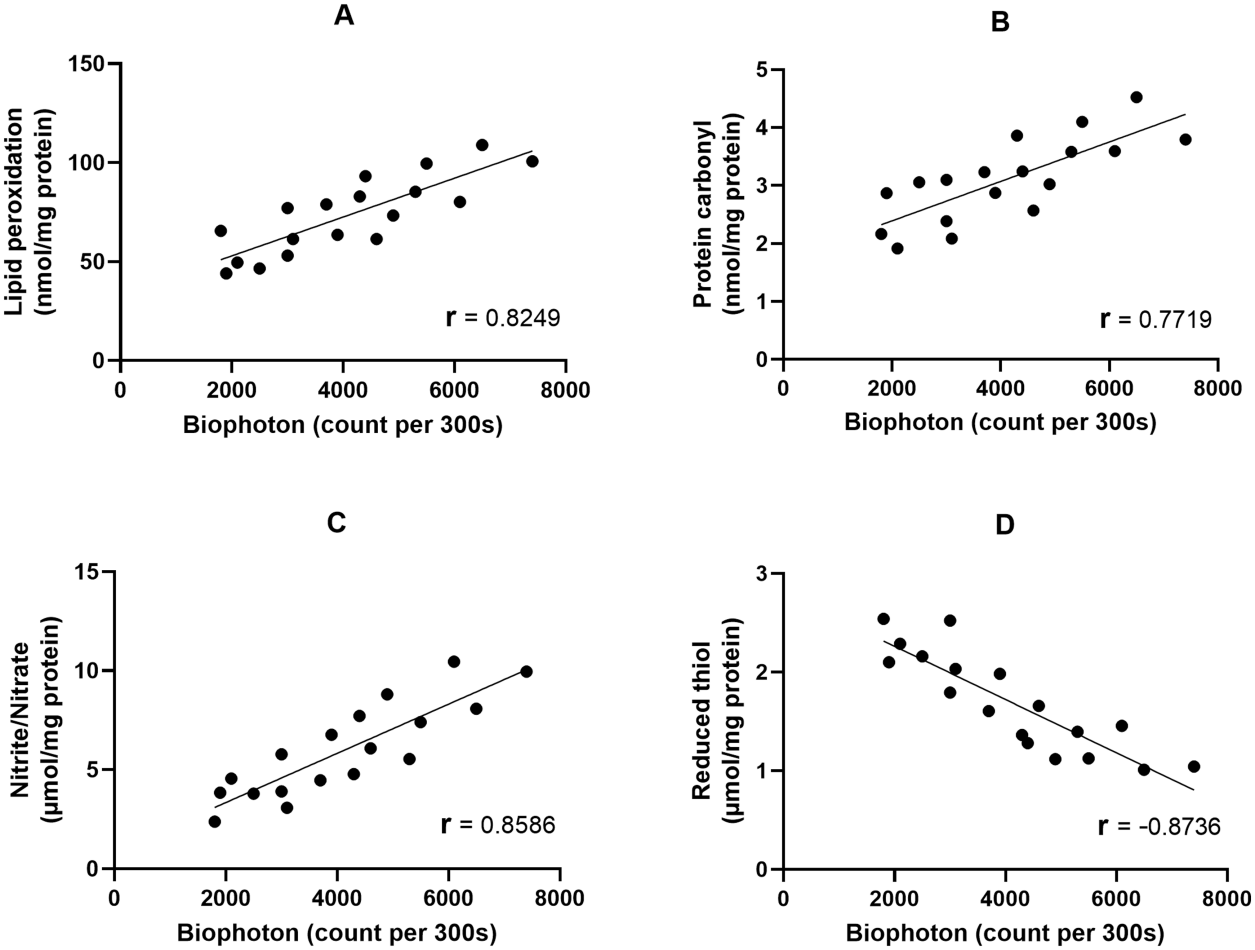
Correlation matrix and linear regression of the isolated brain UPE with oxidative stress markers. **(A)** UPE vs. Lipid peroxidation; **(B)** UPE vs. Protein carbonyl; **(C)** UPE vs. Nitrite/Nitrate; **(D)** UPE vs. Reduced thiol.

The purpose of this study is to evaluate the isolated brain’s UPE (ultraweak photon emission) under anesthesia induced by ketamine and thiopental. The findings indicate that anesthesia induction can indeed alter the UPE of the isolated brain. To further explore the relationship between changes in UPE and the brain’s oxidative-nitrosative stress and antioxidant capacity, we conducted data analysis using Pearson correlation analyses. These analyses showed positive relationships between UPE and lipid (Figure 4A) protein (Figure 4B), and nitrogen (Figure 4C) oxidation of the brain (*r* = 0.8249, *r* = 0.7719, *r* = 0.8586, respectively). Moreover, a negative relation between UPE and the reduced thiol level of the brain was detected (*r* = −0.8736; Figure 4D). UPE had the strongest correlation with reduced thiol level and the weakest correlation to protein carbonyl. The Coefficient of determination (r^2^) indicated that 76% of the UPE variance could be explained by reduced thiol, while 59% of UPE variance could be explained by protein carbonyl.

In agreement, it was shown that the intensity of rat brain UPE is correlated to oxidative stress which arises from electrical activity and energy metabolism (Kobayashi et al., 1999). Studies have demonstrated that ROS and RNS through the oxidation of lipid, protein, and nucleic acid produce excited species. Electronic relaxation of these species appears to be the source of UPE. Mitochondria are thought to be the main source of this activity. Since the respiratory chain of mitochondria contains a large amount of molecular oxygen it is predisposed to radical generation. Therefore, photon emission intensity is closely coupled to oxidative metabolic activity (Pospíšil et al., 2019; Turrens, 2003).

Neuron firing and communication consume ATP, and, hence, scale-up metabolism. Glutamate is the main excitatory neurotransmitter in the CNS. Tang and Dai (2014a, 2014b), showed that the glutamate application on brain slices intensified biophotonic activities and this activity could be blocked by oxygen and glucose deprivation. Ketamine and thiopental have contrasting effects on glutamate release and brain metabolism. It was shown that ketamine through blocking the NMDA receptors on inhibitory interneurons, leads to excitatory activity and glutamate release from downstream pyramidal neurons (Moghaddam et al., 1997). A recent study demonstrated that an anesthetic dose of ketamine broadly enhanced brain metabolism in SD rats, which makes sense given that cerebral metabolism and neuronal activity are connected (Chen et al., 2022). In contrast, it was found that the spontaneous and KCl-evoked release of glutamate in the prefrontal cortical synaptosomes was suppressed by thiopental and it reduced the extracellular level of glutamate in the rat prefrontal cortex (Hongliang and Shanglong, 2004; Liu and Yao, 2005). ^13^C magnetic resonance spectroscopy of rat cortex under anesthesia with thiopental showed a significant decrease in the oxidative metabolism of neurons and astrocytes (Sonnay et al., 2017). As biophotonic activity correlated to glutamate and brain metabolism, consistent with aforementioned evidences, we found ketamine increased the brain UPE while thiopental decreased it (Figure 1).

Ketamine by antagonizing NMDA receptors on interneurons reduces the inhibitory effect of them on monoaminergic neurons. The absence of this inhibitory input leads to an increase in dopamine, serotonin, and norepinephrine release (Dawson et al., 2013; Del Arco et al., 2008; Tao and Auerbach, 1994). On the contrary, it has been shown that thiopental decreases the release of dopamine, serotonin, and norepinephrine (Tao and Auerbach, 1994; Lesser et al., 1999; Lambert et al., 1996).

It was proposed that increased UPE production and decreased UPE absorption and transfer may cause cellular oxidative damage (Kurian et al., 2017). Hence, both ROS and UPE could lead to an increase in oxidative damage. ROS is generated by the interaction of superoxide anion with other molecules. This anion is created by electron addition to molecular oxygen in the respiratory chain of mitochondria and Complex I is the major source of this activity (Murphy, 2009). Venâncio et al. (2015) showed that an anesthetic dose of ketamine disrupted Complex I function, leading to elevated oxygen consumption, reduced efficiency of oxidative phosphorylation, enhanced H_2_O_2_ generation, and nitric oxide synthase by mitochondria. They suggested that this occurred due to reversed electron transport, which is linked to the generation of superoxide and nitric oxide. Consistent with this evidence previous studies showed that shortly after a single dose of ketamine, lipid peroxidation, nitrite content, protein carbonyl, SOD activity, and CAT activity of the brain increased, while the reduced thiol level decreased (Venâncio et al., 2015; Chiu et al., 2015; Schimites et al., 2020; Da Silva et al., 2010). In agreement, we found these changes in the ketamine group (Figures 2, 3).

Increased antioxidative enzyme activity in the ketamine group may result from increased expression of them due to NO-induced extracellular-regulated kinase 1/2 (ERK) pathway or H_2_O_2_-induced translocation of the nuclear factor κB (NFκB) into the nucleus (Scorziello et al., 2007; Storz et al., 2005). A study found that the expression of stress proteins such as SOD1 increased shortly after exposure to oxidative stress (Vogel et al., 2011).

A study that labeled neurons with fluorescent probes found that ketamine reduced mitochondrial membrane potential, increased ROS production, and led to cytochrome c release from mitochondria. Additionally, it demonstrated the differential expression of oxidative stress-related genes, such as SOD1, induced by ketamine (Bosnjak et al., 2012).

A study using immunofluorescent staining confirmed that ketamine treatment resulted in increased expression levels of glucose transporter 3 (GLUT3) and phosphorylated ERK1/2 (P-ERK1/2) in astrocytes, but not in neurons, within the prefrontal cortex and suggested that ketamine enhances glucose metabolism in the prefrontal cortex through the nuclear localization of P-ERK1/2 in astrocytes, which promotes the expression of GLUT3 (Ouyang et al., 2021).

Immunofluorescence staining of a human microglial cell line shows the nuclear translocation of the signal transducer and activation of transcription 3 (STAT3) after the cells were treated with ketamine. This study showed that STAT3, through interaction with eukaryotic elongation factor 2 (EEF2), increases protein synthesis (Ho et al., 2019). Protein synthesis was identified as the most ATP-consuming process in mammalian cells (Buttgereit and Brand, 1995). Thiopental was demonstrated to inhibit global protein synthesis in neurons by inactivating eukaryotic elongation factor 2. Through this mechanism, it preserves ATP and prevents damage in oxygen-deprived cells (Schwer et al., 2013). This evidence could explain reduced SOD and CAT activity in the thiopental group (Figures 3B,C). NF-kB regulates the expression of stress-related genes, and it has been shown that thiopental inhibits NF-kB activation in experimental murine brain inflammation (Ichiyama et al., 2001).

Protein synthesis inhibition by thiopental may also block the translation of inducible nitric oxide synthase, cyclooxygenase-2, or matrix metalloproteinases. These proteins have been implicated in peroxynitrite formation, lipid peroxidation, and protein oxidation (Chatterjee, 2016). In agreement, we found a decrease in lipid peroxidation, protein carbonyl, and nitrite/nitrate levels in the brain of the thiopental group (Figure 2). Research has demonstrated that thiopental inhibits the production of nitric oxide in the aorta of rats (Castillo et al., 1999). Previous studies have demonstrated that thiopental exhibits a potent capacity to scavenge all types of radicals and could prevent lipid peroxidation in cultured neurons following exposure to oxygen and glucose deprivation (Inoue et al., 2023; Almaas et al., 2000). These effects may be the result of the sulfhydryl group present in dissolved thiopental. Indeed, we observed an increase in the levels of reduced thiol in the thiopental group (Figure 3A).

Let us note that anesthesia and UPE are both very interesting also from the perspective of quantum biology. Several quantum mechanisms underlying anesthesia have been suggested, motivated partly by the observation of microtubule-related (Craddock et al., 2017) and quantum spin-related isotope effects (Li et al., 2018), where the latter may be due to the radical pair mechanism (Smith et al., 2021). The radical pair mechanism is a leading explanation for biological magnetosensitivity (Zadeh-Haghighi and Simon, 2022), including many reports of magnetic field effects on reactive oxygen species (Kinsey et al., 2023), which also play a key role in UPE. There are thus multiple reasons to expect that the effects observed in this paper might be magnetic field dependent or isotope-dependent (Zadeh-Haghighi and Simon, 2023), and exploring this question is an interesting avenue for future studies.

## 5 Summary and outlook

This study was on the differential effects of ketamine and thiopental anesthesia on ultraweak photon emission (UPE) and oxidative-nitrosative stress in rat brains. To our knowledge, no previous study has investigated and compared UPE in the presence and absence of anesthesia. From the obtained results one could conclude that the contrasting effects of ketamine and thiopental on UPE intensity are strongly correlated to the brain’s oxidative stress and antioxidant capacity. These findings may contribute to a broader understanding of how anesthetics influence not only physiological parameters but also the subtle bioenergetic and redox dynamics in neural tissues (Salari et al., 2015; Esmaeilpour et al., 2023).

Further research is essential to investigate the effects of other anesthetics on the brain. Moreover, exploring the molecular mechanisms underlying the action of the anesthetics, e.g., the role of mitochondria in anesthesia, could provide a deeper understanding of the role of oxidative stress and UPE as biomarkers for anesthetic efficacy and safety. It will provide further insight into how consciousness changes or shuts down during anesthesia, shedding light on the nature of consciousness itself, and help develop safer anesthesia practices, improving patient care and advancing protective strategies for the brain in medical treatments.

## Data Availability

The original contributions presented in the study are included in the article/supplementary material, further inquiries can be directed to the corresponding author MK.
